# All-Round Manipulation of the Actin Cytoskeleton by HIV

**DOI:** 10.3390/v10020063

**Published:** 2018-02-05

**Authors:** Alberto Ospina Stella, Stuart Turville

**Affiliations:** The Kirby Institute, University of New South Wales (UNSW), Sydney NSW 2052, Australia; sturville@kirby.unsw.edu.au or recpt@kirby.unsw.edu.au

**Keywords:** HIV, actin, cytoskeleton, manipulation, inbound, outbound, virus, cell-cell transfer, virological synapse, Rho-GTPases

## Abstract

While significant progress has been made in terms of human immunodeficiency virus (HIV) therapy, treatment does not represent a cure and remains inaccessible to many people living with HIV. Continued mechanistic research into the viral life cycle and its intersection with many aspects of cellular biology are not only fundamental in the continued fight against HIV, but also provide many key observations of the workings of our immune system. Decades of HIV research have testified to the integral role of the actin cytoskeleton in both establishing and spreading the infection. Here, we review how the virus uses different strategies to manipulate cellular actin networks and increase the efficiency of various stages of its life cycle. While some HIV proteins seem able to bind to actin filaments directly, subversion of the cytoskeleton occurs indirectly by exploiting the power of actin regulatory proteins, which are corrupted at multiple levels. Furthermore, this manipulation is not restricted to a discrete class of proteins, but rather extends throughout all layers of the cytoskeleton. We discuss prominent examples of actin regulators that are exploited, neutralized or hijacked by the virus, and address how their coordinated deregulation can lead to changes in cellular behavior that promote viral spreading.

## 1. Introduction

**Figure 1:**
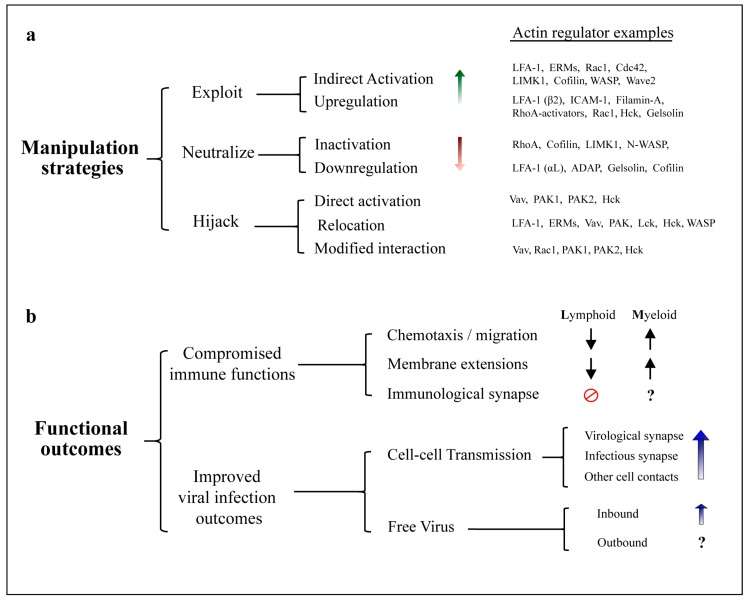
Manipulation of the actin cytoskeleton by human immunodeficiency virus (HIV). (**a**) Actin regulators subjected to modulation by HIV. Mechanistically diverse strategies enable the virus to alter cellular cytoskeletal functions. Manipulation of host factors can be either direct, when mediated by physical interaction with viral proteins, or indirect, when requiring upstream cellular factors. Exploitation mechanisms increase native protein activity by upregulation of gene expression, or indirect activation within a cellular pathway. Neutralization of host factors is achieved by downregulation of gene expression or protein inactivation. Hijacking alters the functional outcome of host protein activity, either by overriding regulatory mechanisms (i.e., direct protein activation), changing protein subcellular localization, and/or modifying protein interaction partners. Note that some host factors can be manipulated by multiple strategies at diverse stages of the viral life cycle, as well as differentially in infected and uninfected cells. Examples of actin regulators corresponding to each strategy are provided, however this is not a complete list; (**b**) Functional consequences of actin-dependent changes induced by HIV. Normal immunological functions are compromised upon HIV infection, partly due to actin-remodeling changes orchestrated by viral proteins. CD4+ lymphocytes display severe impairment of chemotaxis and immunological synapse formation. Myeloid cells display aberrant enhancement of actin dependent structures, which alters cell motility and tissue distribution. Concurrent changes in actin remodeling in both cell types also promote viral spread via actin-dependent cell-cell contacts and support infection by inbound cell-free virus.

Actin is the most abundant protein in human cells and is essential for a wide range of cellular processes [1], including transport of cargo and maintenance of cellular architecture. Intracellular pathogens are therefore obliged to navigate through and interact with the host cell’s actin cytoskeleton. The key importance of this ability is emphasized by the fact that mechanisms to manipulate cellular actin networks have convergently evolved across incredibly diverse pathogen families, including most human viruses [2,3] and many species of bacteria [4]. While many pathogens encode specific proteins that mimic cellular regulators in order to directly mediate actin remodeling [5], none of the known human immunodeficiency virus (HIV) gene products seems to be dedicated to this task. Instead, HIV manipulates actin dynamics indirectly by leveraging many of its essential components to exercise control over a wide range of cytoskeletal regulators and pathways. This multi-strategy and multi-target approach makes manipulation of cellular actin networks by HIV particularly complex to study. Observations from the literature report that the virus can exploit, neutralize or hijack cytoskeletal proteins, by promoting protein activation/inactivation, modulation of gene expression, changes in cellular localization and even altering cellular protein function, e.g., by modifying protein interaction partners (Figure 1). Some host factors are even manipulated by multiple strategies, depending on the stage of the viral life cycle. Far from being random in nature, these manipulation events are spatiotemporally regulated, and their coordinated implementation results in cell-type specific changes in cellular behavior to promote defined outcomes which are overall favorable for HIV infection. Most notably, these include impairment of immune cell functions and improvement of viral outcomes both in terms of cell-free virus and cell-cell spread (Figure 1).

Subversion of the host actin cytoskeleton is essential for both establishing and spreading the infection by HIV. Virtually all known stages of the viral life cycle have been reported to be dependent on actin to lower or greater extent, including viral entry [6,7,8], reverse transcription [9], nuclear migration [10,11,12], transport of viral components to the membrane [13,14,15], assembly [16,17,18], budding [19,20,21] and cell-cell transfer (see Section 3). Of note, the microtubule network has also been identified to play several roles during HIV infection; however, this extends beyond the scope of this review (we refer to [17,22,23,24]). Early studies identified a general role of actin filaments (F-actin or “microfilaments”) in HIV infection mainly by using a broad range of actin-disrupting pharmacological agents (see Section 4.3). Despite the importance of these seminal observations, the cytotoxicity and pleiotropic effects associated with these drugs, as well as their inability to selectively inhibit specific actin-driven cellular processes, underscores the need for a better understanding of the interplay between HIV and cellular actin. More recently, advances in technologies such as genetic manipulation and high-resolution microscopy have demonstrated the involvement of numerous cytoskeletal proteins, which act upstream of microfilaments during HIV-induced actin remodeling. Other authors have previously addressed how HIV takes advantage of certain actin-related pathways, with emphasis on viral entry and early steps of the viral life cycle [22,25]. In this review, we provide an updated overview of how HIV employs different strategies to exercise control over a wide range of host proteins throughout all levels of the actin cytoskeleton. By incorporating the latest findings and considering important differences between cell types, we consolidate available knowledge regarding the roles that individual actin regulators and whole pathways play at particular stages of HIV infection. Whenever possible, we highlight this in the context of cell-cell viral transfer, given its now recognized role as the predominant mode of HIV spread (see Section 3).

## 2. Inbound vs. Outbound HIV

When considering manipulation of actin by HIV, it is important to distinguish between events occurring in cells which are already productively infected, and those in uninfected cells upon challenge with virus. We define “inbound virus” as any naturally occurring infectious particle that originated from an infected cell and which, after an extracellular phase of variable length, is in the process of moving from the plasma membrane (PM) to the nucleus of a different cell. This includes mature virions bound to target cells, and the intermediates which result after membrane fusion and during uncoating, reverse transcription, nuclear transport and genome integration. In contrast, we define “outbound virus” as the collection of viral components which are produced de novo in an infected cell and which are in the process of exiting this cell in the form of initially immature HIV particles. This includes viral proteins and RNA during their transport to the PM, particle assembly and budding, up to the point of abscission. An important difference between the two is that manipulation of the cytoskeleton by inbound virus can only rely on viral factors that are incorporated in the virion (mainly proteolytically cleaved mature products of Gag and Gag-Pol, alongside Vpr, HIV envelope glycoprotein (Env) and Nef), and is limited by the relatively low abundance of these proteins, compared to the large pool of host cytoskeletal proteins in the target cell. In contrast, during outbound virus production, viral RNA and proteins (including all accessory proteins) are present at high concentrations in the cytosol and, therefore, have direct access to cytoskeletal components, most of which are purely intracellular. A third scenario is given by soluble viral proteins such as Tat, Env and Nef, which are known to be present extracellularly, as well as inside uninfected cells both in vitro and in vivo [26,27,28]. Thus, these soluble factors can induce cytoskeletal changes in a broad range of cells, including cell types that are not typically infected by HIV. Of note, our definition of inbound and outbound HIV extends to both cell-free and cell-cell transmitted virus.

## 3. Cell-Cell Transfer of HIV

Both free virions and several types of infected cells are present in the blood and tissues of untreated HIV+ individuals. Given the high plasma viral loads present during acquired immune deficiency syndrome (AIDS), HIV spread was initially thought to be mediated only by free viral particles, which could travel long distances within the organism. However, it was recognized over two decades ago that the virus can also spread from donor cells via direct cell-cell contacts [29,30,31,32]. In the last 25 years, a vast amount of evidence has accumulated and revealed cell-cell transfer of HIV to be more efficient than infection by cell-free virus in virtually every study which directly compared both, at least in vitro. This observation has gained further support from in silico [33,34], ex vivo [35,36], and in vivo studies [37,38,39,40,41,42], so that cell-cell transfer of HIV is now accepted by the field as a major mode of viral spread [42,43,44,45,46]. An integrated view is provided by Zhang et al., who combine patient data and mathematical modelling to conclude that both cell-cell and cell-free virus are required for HIV pathogenesis. However, even this model predicts that while “hybrid spreading” is fundamental for establishing initial infection, cell-cell spread becomes increasingly more effective as the infection progresses [34]. Importantly, cell-mediated transfer of HIV is strongly dependent on actin remodeling [17,47,48,49,50], given the role of actin filaments for: (i) membrane protrusions involved in intercellular contacts; (ii) formation of stable conjugates; and (iii) polarization of both donor and target cells. In this section we introduce the two major structures involved in cell-cell transmission of HIV, and in Section 5 we address the known roles of different cytoskeletal players in their formation and regulation.

### 3.1. The Virological Synapse

The virological synapse (VS) can be defined as an organized supramolecular structure that forms at the contact area between a virus-infected cell and an uninfected target cell, with concomitant polarization of viral effector and receptor molecules at the donor-target cell interface, respectively, and which mediates direct cell-to-cell viral transfer in the absence of cellular fusion [43,48,51,52,53,54]. Such transfer of viral material that was synthesized de novo in the productively infected donor cell is referred to as transfer “in cis” [55]. The hallmark feature of the HIV-VS is actin-dependent co-polarization of Env and CD4 [48]. However, it is important to note that VS can form between different cell types, leading to distinct synapse architectures [42,56], and sometimes diverse roles for the numerous cytoskeletal players involved. While the homotypic T-cell variant is by far the best understood type of VS [51], some progress has been made regarding VS that form between infected myeloid cells and target T-cells [57,58,59,60,61].

### 3.2. The Infectious Synapse

A special scenario is presented by cells which, without themselves becoming productively infected, can capture virus and transfer it to target cells “in trans” (reviewed in [55]). Contacts between such “carrier” cells and uninfected T-cells that result in viral transfer are known as infectious synapses. The canonical infectious synapse occurs between HIV-loaded dendritic cells (DC) and T-cells [49,62], and involves extensive intercellular contacts mediated by specialized membrane protrusions [63,64]. However, other infectious synapse variants continue to be discovered, with monocytes, macrophages, B-cells and even fibroblasts being able to take up HIV virions and subsequently infect target cells in trans [36,42,55,65,66,67].

## 4. Direct Association between HIV and F-Actin

### 4.1. Actin in HIV Virions

The presence of actin in enveloped viruses was recognized over four decades ago [68], yet a functional role within the virions (if any) remains unresolved. In 1996, Ott et al. reported the presence of actin within HIV produced in T-cell lines, exceeding 10% of the molar abundance of Gag within the virion [69]. Gag alone was then shown to be sufficient for actin incorporation into the particle [70]. Several groups have confirmed that both actin and numerous host cytoskeletal proteins are incorporated within HIV virions [71,72,73]. A conserved set of host proteins shared by virions produced in T-cells and macrophages was identified by Linde et al., with about half corresponding to actin isoforms [73]. Both the mechanisms by which cytoskeletal proteins get incorporated and their potential roles within viral particles remain mostly controversial [74,75], with few exceptions. Nevertheless, it is well accepted that identification of these host factors can provide important information regarding potential viral–host protein interactions, the microenvironment of viral assembly and the cellular origin of virions. A recent study has challenged the view of active packaging of actin into HIV particles. Stauffer et al. reported the load of actin within virions to be lower than previously described, and postulated that this parameter is affected by the concentration of actin in the cell of origin. Thus, actin in virions might be simply due to the pinching of cytosolic pools during budding [76]. However, this interpretation is currently limited by the technical inability to measure actin concentrations at different subcellular regions, i.e., at HIV budding sites.

### 4.2. Physical Interaction between Gag and F-Actin

In addition to their ability to manipulate actin regulators, some HIV proteins might be able to associate with actin filaments directly [9,77,78,79]. The structural protein Gag represents the best understood example in this respect. Early studies showed that Gag cosediments with the cytoskeletal cellular fraction and that this association could be disturbed by actin- but not microtubule-depolymerizing drugs, thus suggesting a direct physical interaction between HIV-Gag and microfilaments [80]. Soon after, the nucleocapsid domain of Gag (NC) was observed to be the one required for its association with F-actin. Absence of NC, but not matrix or capsid, abrogated cofractionation of Gag with the cytoskeleton, whereas recombinant NC was able to bind polymerized F-actin in a dose-dependent manner in vitro. Furthermore, Gag could be coimmunoprecipitated with actin but not tubulin. Based on these observations, a direct physical interaction between HIV-Gag and F-actin via Gag’s NC-domain was concluded [81]. Immunogold labelling and electron microscopy also showed that actin significantly colocalizes with NC within the virion. Even after detergent and protease treatment, about half of the virion-associated actin content remained in stable complex with NC [70]. Given the importance of viral RNA binding to NC during the viral life cycle, investigations of NC-actin binding and how this is influenced by viral RNA should be further explored. A specific and intimate physical association (<5 nm) between HIV-Gag and actin was confirmed by fluorescence resonance energy transfer (FRET), albeit in fixed and adherent cell lines [82]. Overall, convincing evidence supports physical association between HIV-Gag and F-actin, although whether or not this interaction plays any function role in the viral life cycle is still unknown.

### 4.3. Role of Actin for HIV Entry

**Figure 2:**
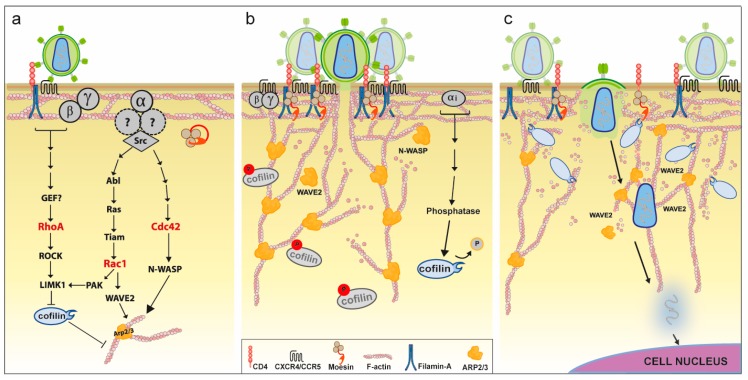
Hypothetical model of the role of manipulated actin dynamics for inbound HIV infection in T-cells. (**a**) Early signaling events. Binding of Env to CD4 and coreceptor molecules (CXCR4/CCR5) initiates signaling via the receptor-coupled G-protein. Gα_i_/Gα_q_ signaling via Src activates both Rac1 and Cdc42, which in turn activate downstream nucleation-promoting factors (NPFs) that stimulate actin polymerization activity by the Arp2/3 complex. Env binding to CD4 also triggers RhoA activation in a filamin-A-dependent manner, and results in moesin activating phosphorylation. Both RhoA and Rac1 lead to cofilin inactivation via phosphorylation by LIMK1. Rho-GTPases are shown in red; (**b**) receptor capping. Stimulation of Arp2/3 by active NPFs and simultaneous inhibition of cofilin leads to increased cortical actin polymerization. This results in aggregation of CD4 and CXCR4 receptors, which requires linking of these molecules to actin filaments by filamin-A and moesin. Enhanced colocalization of these receptors near Env promotes viral entry (fusion), with actin polymerization participating in fusion-pore formation and expansion. Delayed Env-CXCR4 signaling via Gα_i_ leads to cofilin activating dephosphorylation by a currently undefined mechanism; (**c**) intracellular transport. After virus–cell fusion, transport of HIV to the nucleus is limited by the dense cortical actin network. Cofilin activation results in local actin depolymerization that allows passage of the viral core through the actin cortex. Transport of the pre-integration complex towards the nucleus is thought to involve Arp2/3-mediated actin polymerization. * Of note, interfering with the Rho-GTPases shown in Red, or any of the molecules depicted in the legend has been reported to impair inbound HIV infection.

Several observations following the use of actin-disrupting drugs indicated an essential role of actin remodeling for inbound HIV. Inhibitors of actin polymerization dramatically decrease HIV infection in T-cells, by arresting viral entry at a pre-fusion step [6,83,84,85]. A seminal study by Iyengar et al. revealed that Env induces actin-dependent concentration of CD4 and CXCR4 receptors, which become copolarized on the target cell surface [6]. This is thought to increase the density of viral receptors in the vicinity of membrane-bound HIV virions and thereby promote viral entry. In contrast, treatment of virions with cytochalasin-D did not reduce their infectivity [6], indicating that only actin changes in the target cell are required. Subsequent studies identified many of the cytoskeletal regulators involved in this process and confirmed that actin remodeling is required for viral entry both before and after membrane fusion. Unlike for other stages of the viral life cycle, sufficient evidence exists to provide a relatively complete and unambiguous model. Figure 2 summarizes our updated view of the molecular mechanisms involved in actin regulation around HIV entry (as previously proposed by [86,87]). The mechanisms by which HIV manipulates cellular proteins to orchestrate these events in T-cells are also further discussed in detail in Section 5. Furthermore, a critical role of actin for HIV entry has been recently confirmed in macrophages [8].

### 4.4. Role of Actin for Free-Virus Egress

While the importance of cellular actin-remodeling for cell-cell transmission is relatively undisputed (reviewed in [2,17,22,25]), the role of actin filaments towards release of outbound free viral particles remains controversial. Studies using actin-disrupting drugs have so far reported inconsistent effects on viral release. The actin polymerization inhibitor cytochalasin-D either reduced or had no effect on HIV release [17,20,21,88]. While this might be explained by differences in cell lines and experimental conditions, no unified conclusion can be drawn from these observations. In contrast, complete depolymerization of F-actin by the potent marine toxin mycalolide-B decreased HIV release in a dose-dependent manner, with viral proteins failing to reach the PM [14]. In addition, several studies using highly sensitive methods have observed prominent association of HIV budding sites with F-actin. Using atomic force microscopy, Gladnikoff et al. visualized aster-shaped, NC-dependent actin structures that emanate from HIV buds during viral assembly and disappear upon viral release [18]. Cryo-electron tomography confirmed the presence of actin filaments near budding sites, and even revealed occasional and direct physical contacts with nascent particles [18,76,89]. However, some observations argue against a functional role of these actin filaments in viral budding. Firstly, the absence of NC did not reduce actin content near budding sites [76]. Secondly, real-time tracking of single-virus buds revealed no F-actin recruitment during viral assembly [90]. This recent study also showed that budding-initiation and Gag assembly were not affected in the presence of actin dynamics inhibitors, despite confirmed disruption of the cortical F-actin network [90]. However, since the latter studies did not measure supernatant viral contents, a role for actin in the actual egress of HIV virions cannot be excluded. Indeed, studies looking at particle production upon interference with prominent actin regulators do suggest a role of actin dynamics in HIV budding ([19,91] and see below).

## 5. Manipulation of the Actin Cytoskeleton

**Figure 3:**
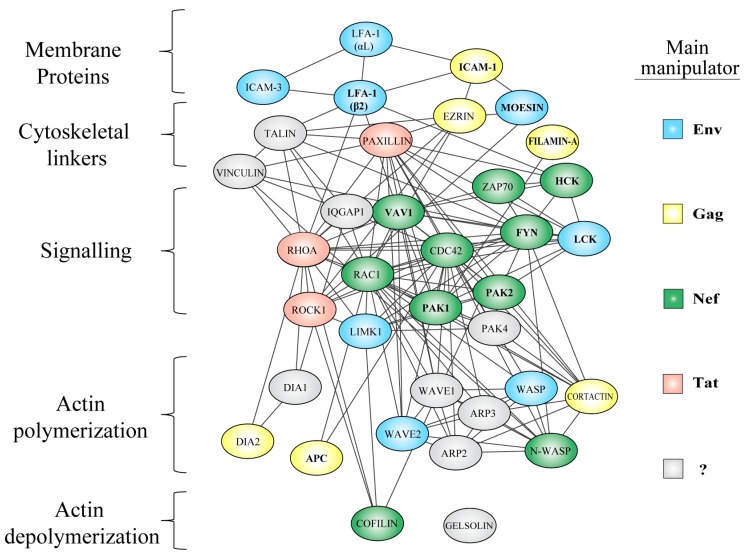
Functional protein interaction network of actin regulators targeted by HIV manipulation. Subversion of the actin cytoskeleton occurs throughout all layers of the actin cytoskeleton, including surface proteins, their linkers to actin filaments, diverse signaling nodes, their effectors, and proteins that directly mediate actin remodeling. Color coding of host factors indicates the main viral protein involved in their deregulation; Nef = green, Gag = yellow, Tat = pink, Env = blue, presently undefined = grey. So far, only proteins shown in bold have been experimentally confirmed to physically interact with the indicated viral proteins (i.e., direct manipulation). Network data was obtained from the STRING database [93] using the following settings: network edge meaning = evidence, active sources = databases, minimum confidence score = 0.9, number of interactors = query only (proteins covered in this review). The network nodes were rearranged using Cytoscape software [94], to represent functional groups within the cytoskeleton.

While HIV can directly associate with F-actin, the most striking manipulation of the cytoskeleton occurs by reprogramming the expression, activity and interactions of actin-regulatory proteins upstream of microfilaments (see Figure 1). In this section we review prominent examples of regulators that are exploited, neutralized or hijacked by HIV, and discuss how their manipulation at precise stages of the viral life cycle promotes specific viral infection outcomes. We also emphasize that manipulation is mechanistically diverse and not limited to a particular class of proteins, but rather extends throughout all layers of the cytoskeleton (Figure 3). It should be noted, however, that cytoskeletal regulation is highly cell-type specific, and this is evidenced by the clear differences in cell morphology and motility of heterotypic cells, which are largely given by differential expression patterns of actin regulatory proteins. Therefore, it is important to consider this heterogeneity while discussing manipulation of the actin cytoskeleton, because there are often aspects that are cell-type-specific and/or observations in cells for which HIV has no natural tropism (i.e., cells engineered or transfected to express viral receptors). Thus, here, we have aimed to inform on the cell systems used in the individual studies to consider this layer of complexity. Relevant human cell lines commonly used in the study of HIV and actin include the T-cell lines Jurkat, CEM and H9, as well as the monocytic cell lines U937 and THP1. In addition, many mechanistic studies have used adherent cells such as the human embryonic kidney cell line HEK 293T, and the epithelial cervical carcinoma cell line HeLa. Note that from here on we only use the term “T-cells” to refer to observations based on data that also includes primary CD4+ lymphocytes. The role of many of the proteins presented here as therapeutic targets for HIV treatment is partially discussed in [92].

### 5.1. Exploitation of Actin Regulators and Pathways by HIV

While some HIV proteins can physically engage cellular actin regulators and directly activate them, a greater number of host factors seems to be taken advantage of indirectly. Their ability to promote specific changes in the actin cytoskeleton can be exploited by HIV, even in the absence of direct physical interaction, by increasing total protein abundance or native protein activity within cellular pathways. We consider host factors to be exploited by HIV whenever this increase occurs by mechanisms that do not affect endogenous protein function or subcellular localization. Based on the available knowledge, this so far includes; (i) upregulation of gene expression; and (ii) indirect protein activation (i.e., downstream of host factors that are directly activated by HIV proteins). Since host factors are often differentially manipulated in infected and uninfected cells, as well as in different cell types, actin regulators that are exploited by HIV at a given step of the viral life cycle may also be neutralized or hijacked at other stages during the course of HIV infection (Figure 1a). For the examples discussed here, we first address how each factor is exploited by inbound HIV and then consider how manipulation by outbound HIV aids viral egress and/or cell-cell transmission. The mechanism(s) enabling such exploitation are discussed where there are sufficient observations to build a hypothetical model.

#### 5.1.1. Membrane-Cytoskeleton Linkers

Membrane-cytoskeleton linkers are proteins associated with the inner leaflet of the PM, which can bind both surface-proteins and F-actin directly [95]. They can mediate anchorage, docking and movement of membrane proteins along the cell surface, while also serving as scaffolding proteins to facilitate signal transduction by engaged membrane receptors.

**Filamin-A** can crosslink actin filaments in wide angles to form orthogonal actin networks at the cell cortex [96], and regulates cell migration and adhesion via integrin modulation [97]. Filamin-A is exploited by inbound and outbound HIV to promote both viral entry and release. In uninfected T-cells, HIV entry was shown to be dependent on filamin-A [98]. The interaction between filamin-A and CD4 was strictly required upon Env-CD4 binding for RhoA-ROCK1-LIMK1-signalling, which mediates cofilin inactivation (see Figure 2a and Section 5.1.4). This enables actin polymerization and recruitment of CD4 and CXCR4 molecules to the contact interface to facilitate viral entry. Silencing (siRNA/shRNA post-transcriptional silencing) of filamin-A or disrupting its interaction with CD4 in target cells strongly reduced receptor co-capping and infection by inbound cell-free virus [98]. On the other hand, filamin-A is one of the highest upregulated genes upon HIV infection, as evidenced by dramatically increased protein levels in infected T-cell lines (ten-fold in Jurkat cells, and within 10% of up-regulated proteins in CEM cells) [15,99], and in PBMCs of HIV+ patients [100]. The findings of recent studies suggest that increased filamin-A levels likely boost outbound viral release from infected cells by binding to Gag and increasing its trafficking to the PM. Filamin-A overexpression increased particle release by three-fold in Jurkat cells, whereas depletion in HeLa cells impaired localization of Gag to the PM and reduced viral release two-fold [15]. Furthermore, filamin-A was shown to specifically interact with HIV-Gag in vitro, and to colocalize with membrane Gag in HeLa cells [15]. An intimate association between filamin-A and outbound Gag was confirmed by a recent study, which identified filamin-A as the most biotinylated cellular protein when a T-cell line was infected with virus encoding biotin-ligase-tagged Gag [101]. Therefore, filamin-A incorporation into HIV virions [102], might be a consequence of its physical interaction with Gag. In conclusion, filamin-A is required for HIV entry into target cells, whereas in infected cells, it is strongly upregulated and promotes outbound viral release. However, since no changes were detected at the transcriptional level [99], the mechanism by which HIV increases filamin-A abundance, and the identity of the responsible viral factor remain important unresolved questions in the field.

**ERMs** (Ezrin, Radixin and Moesin) are a family of highly homologous membrane-linker proteins that participate in diverse cellular processes including actin organization, cell migration and survival [103]. They are commonly found in membrane protrusions such as filopodia, microvilli, and membrane ruffles. Various studies suggest a key role for moesin during inbound HIV infection. Silencing of moesin reduced HIV infection in T-cells at a pre-fusion step [104,105]. Barrero et al. showed that Env-CD4 interaction was followed by moesin activating phosphorylation and clustering of F-actin, CD4 and CXCR4 in a moesin dependent-manner [104]. This suggests that inbound HIV activates moesin via Env to link these proteins to actin filaments thus enabling receptor capping, which in turn promotes viral entry (Figure 2b). In contrast, the role of ERMs for outbound virus is less well understood. HIV infection has been shown to increase expression of moesin or ezrin in various cell types, including CEM, HeLa and monocytes [106,107,108]. However, silencing of either protein increased HIV replication in infected Jurkat cells [107,109], suggesting a rather restrictive role of ERMs in outbound HIV spread. Still, both proteins have been consistently found within HIV virions, where they are specifically incorporated and reach up to 2% of the molar abundance of Gag [69,73,102]. Since recent studies identified ezrin as a putative binding partner of Gag [109], and reported a positive role for ezrin in virion infectivity [110], we believe this could explain how and why this host protein is incorporated into HIV particles. Overall, both ezrin and moesin seem to be important targets of manipulation by HIV, but further work will be required to fully define their contributions to outbound HIV spread.

**Focal adhesion proteins** (talin, vinculin and paxillin) link integrins and other surface adhesion molecules to cortical actin filaments and are, therefore, essential components of cell-matrix and cell-cell contacts [111,112]. Both talin and vinculin expression is up-regulated in PBMCs from HIV-infected patients [100,113]. This is interesting because a later study has reported a rather restrictive role of these proteins for inbound free-virus infection. Overexpression of either protein inhibited early post-fusion steps of HIV infection, whereas their depletion led to increased infection. However, these effects were observed in cell lines of non-hematopoietic lineage and were independent of actin-remodeling [114]. Based on these observations, we hypothesize that selective upregulation of focal adhesion proteins in productively HIV-infected cells might promote outbound virus spread (e.g., by enhancing virological synapse formation), whereas in uninfected cells, they might serve as restriction factors for inbound HIV. This is supported by the fact that vinculin upregulation was limited to HIV-infected cells [113], whereas no conclusion can be drawn for talin in this respect [100].

#### 5.1.2. Rho-GTPase Signaling

Rho-GTPases are small (≈21 kDa) guanine nucleotide-binding proteins that can act as molecular switches and orchestrate many actin-dependent processes including cell morphology and migration [115]. Rac1 and Cdc42 belong to this family of “master regulators of the cytoskeleton” [116], and they represent two of the proteins most potently and diversely manipulated by HIV, despite a clear lack of evidence to suggest direct interaction with viral proteins.

**Rac1** is known to regulate several actin polymerization pathways, which in healthy cells allow formation of membrane ruffles and lamellipodia. Rac1 has been shown to play a determinant role for inbound HIV infection. Harmon et al. elegantly showed that HIV triggers Rac1 activation via Env to promote actin polymerization events that support viral entry (Figure 2a). This is dependent on the Env-CCR5 interaction, which signals over a defined Gαq cascade and results in activation of the tyrosine kinase Abl [7,117]. Abl then phosphorylates Ras, allowing it to stimulate Tiam1, which is a potent GDP exchange factor (GEF) and activator of Rac1 [7,118]. Both Rac1 and Abl activate WAVE2 to prompt actin polymerization by the Arp2/3 complex (see Section 5.1.3). Since disruption of any of these proteins resulted in decreased HIV entry and arrested fusion at the hemi-fusion stage, the authors conclude that this signaling pathway ultimately leads to actin remodeling changes that facilitate HIV fusion-pore formation and enlargement (Figure 2b) [7,108,119]. In agreement, studies by Pontow et al. confirmed that interfering with Rac1 function reduces viral fusion, leading to decreased infection rates [120,121]. While these mechanisms were elucidated mainly in adherent cell lines, other studies support Env-dependent activation of Rac1 in primary T-cells and monocytes ex vivo, as well as brain macrophages in vivo [108]. Of note, soluble Tat is also able to activate Rac1 in uninfected endothelial and HeLa cells, although the corresponding mechanisms are less well understood [122,123,124].

On the other hand, HIV-infected cells also display dramatic Rac1 activation, and this is mainly mediated by Nef. Nef expression increased Rac1 activity in a wide range of cell types, including podocytes [125,126], T-cells [127], monocytes [108,128] and DCs [129]. The mechanism of Nef-mediated Rac1 activation is currently controversial but most likely involves direct binding of Nef to the leukocyte-enriched GPD-exchange factor Vav1 (see Section 5.3.1) [129,130]. However, Nef was also shown to dramatically increase Rac1 expression in podocytes [126], suggesting multiple exploitation mechanisms by the same viral protein. In terms of outbound virus, silencing of Rac1 led to decreased viral production in T-cells, but only for Nef-encoding virus [91]. As above, this effect was also mapped to the Rac1-Wave2-Arp2/3 actin polymerization pathway. Interfering with any of these proteins was associated with reduced membrane-targeting of outbound Gag and impaired viral release [91]. In agreement, constitutively active Rac1 boosted free-virus release from a fibroblast cell line [131]. It is worth noting that while most Rac1 activation in infected cells is mediated by Nef, the presence of Gag alone also led to strong Rac1 activation and actin polymerization in Jurkat cells [91]. In conclusion, Env-triggered Rac1 signaling and potent Nef-induced Rac1 activation mediate actin remodeling changes that promote both inbound and outbound viral spread, respectively. The fact that Nef, Env, Gag and Tat all independently lead to Rac1 activation in human cells, emphasizes layers of redundancy that enable activation, and in turn highlight this protein as a critical regulator in HIV infection. Importantly, aberrant Rac1 activation also occurs in other deadly human diseases (most notably cancer and cardiovascular disease), so that development of clinically-suitable Rac1 inhibitors continues to be a highly pursued goal with potential implications for the field of HIV therapeutics [132,133,134,135].

**Cdc42** is well known for its physiological role in promoting long and actin-rich membrane protrusions such as filopodia [136]. The role of Cdc42 for inbound HIV has been studied by Swaine et al. and recently reviewed in [137]. Briefly, novel Cdc42-specific inhibitors have revealed that, as for Rac1, Env-triggered Cdc42 signaling mediates actin-remodeling changes that facilitate viral entry. The authors conclude that the Cdc42-NWASP-Arp2/3 pathway may be equal to or more important than the Rac1 pathway described above [137]. Recent studies have confirmed this hypothesis, by showing that Env signaling through CD4 and CCR5 leads to activation of both Rac1 and Cdc42 in primary CD4 T-cells (Figure 2a) [138]. Similarly, ingenuity pathway analysis scored Cdc42 and Rac1 pathways within the top-five deregulated cellular networks in T-cells that are exposed to cell-free [138], or cell-transmitted virus [139].

In terms of outbound HIV, Cdc42 seems to play a negligible role in free-virus-release [91], despite being incorporated into virions produced in both T-cells and macrophages [73,102]. In contrast, Cdc42 plays an important role in cell-cell viral spread. When dendritic cells are exposed to HIV, binding of Env to the DC-specific lectin receptor DC-SIGN triggers rapid activation of Cdc42 downstream of the tyrosine kinase Src [64,140]. Cdc42 then activates various actin polymerization pathways that lead to an increase in membrane extensions (see Section 5.1.3). These actin-rich projections on the surface of HIV-pulsed dendritic cells promote viral transfer to target T-cells via infectious synapses [50]. Interfering with Cdc42 activity or expression significantly reduced both the number of membrane extensions and infection of target cells in trans, whereas overexpression or activation had the opposite effect [50]. Of note, Cdc42 did not directly impact HIV capture or conjugate formation by DCs, but was required for rapid and linear transfer of virions along the surface of dendritic cell (DC) membrane extensions towards target cells [50]. In infected cells, some of the effects mediated by Nef require functional Cdc42 [131]. It was proposed that this is due to Cdc42 activation by Vav [141]; however, some studies have failed to detect such Nef-mediated Cdc42 activation [125,127]. Overall, Cdc42 is subject to strong manipulation by HIV and plays important roles in both inbound and outbound viral spread, by promoting viral entry and cell-cell transfer, respectively. Given the recent development of specific Cdc42 inhibitors [137], elucidation of the mechanisms by which Nef, Env and even Tat [124] engage and exploit Cdc42 signaling would represent major contributions to the field.

Overall, the Rho-GTPases Rac1 and Cdc42 are targets of extensive HIV manipulation, and represent signaling nodes of actin regulation, where multiple HIV-manipulated cytoskeletal pathways converge (Figure 3). In particular, signaling to the Arp2/3 complex and subsequent actin polymerization events seem to be crucial for mediating both viral entry and egress. In agreement, inhibition of Rho-GTPase prenylation by statins was associated with reduced PBMC permissibility to infection both in vitro and in vivo. Statins, which are widely used in treatment and prevention of cardiovascular disease [142], decreased viral loads and increased CD4+ cell counts in animal models and in chronically HIV-1-infected patients [143]. This, and other studies mark an important precedent that highlights the potential role of Rho-GTPases as viable pharmacological targets in HIV therapy [144].

#### 5.1.3. Actin Polymerizing Factors

Rapid reorganization of the actin cytoskeleton is essential for many key cellular processes and requires both actin polymerization and depolymerization to occur in a concerted and orchestrated fashion. It is the combination of active regulators at any given time and subcellular location that determine the functional outcome of such actin remodeling [145]. Actin nucleators are factors that can initiate formation of an actin filament starting from a pool of actin monomers [146]. Cells require the ability to “seed” such actin filaments because spontaneous filament assembly is highly inefficient, as it involves formation of unstable dimer and trimer intermediates [147]. Here, we review recent evidence that implicates a functional intersection of HIV with all three classes of actin nucleators.

**The Arp2/3 complex** is highly conserved among eukaryotes as a major component of the actin cytoskeleton and is involved in numerous critical cellular functions [148]. Two of its seven subunits (Arp2 and Arp3) emulate an actin dimer [149], yet the complex displays little activity by itself until one of many “nucleation promoting factors” (NPFs) completes the actin nucleus (trimer) by providing the third actin monomer. The hallmark feature of Arp2/3 is its ability to create branched actin networks, which are essential for formation of the actin cortex and most membrane protrusions [145]. Several studies have revealed a critical role for Arp2/3 at multiple stages of the HIV life cycle. The role of Arp2/3 downstream of Rac1 and Cdc42 in free-virus entry and egress was discussed earlier in this article (see Section 5.1.2). In addition to these effects, Komano et al. showed that inhibition of Arp2/3 also reduced inbound HIV infection at a post-fusion step, and suggested a potential involvement of Arp2/3 in movement of the HIV viral core through the actin cortex [150]. This was confirmed and expanded by Spear et al., who used several additional methods to inhibit Arp2/3 activity in primary T-cells [11]. As previously discussed, Env-CCR5/CXCR4-Rac1 signaling leads to phosphorylation and activation of the NPF protein WAVE2, which stimulates Arp2/3 actin polymerization. Interfering with Arp2/3 activity in this context strongly reduced inbound HIV infection, despite only moderately reduced entry and unimpaired reverse transcription. Given a dramatic decrease in 2-LTR circles (a correlate for nuclear entry [151]), the authors concluded Arp2/3 and Wave2 to be essential cofactors for HIV nuclear migration (Figure 2c) [11].

On the other hand, Arp2/3 activity is also likely to play an important role in cell-cell transmission of HIV. In HIV-pulsed DCs, activation of the NPF “WASP” via Cdc42 increases its complexing with Arp2/3 and actin, inducing formation of membrane extensions that participate in transfer of virus across infectious synapses [64]. Interfering with Arp2/3 activity impaired infectious synapse formation and T-cell infection in trans [50]. Furthermore, 4 of the Arp2/3 subunits were identified in a shRNA library-screen as factors that promote cell-cell transfer of HIV [152]. Since many intracellular pathogens encode specific regulators that directly manipulate the Arp2/3 complex and highly enhance cell-cell transmission [5,153], manipulation of this important host factor by HIV should continue to be explored in this context.

**Formins** act downstream of Rho-GTPases to mediate a broad variety of functions [154], by regulating both actin and microtubule dynamics [155,156]. Actin nucleation by formins leads to polymerization of unbranched linear actin filaments by a complex mechanism [146,157,158]. Two diaphanous-related formins (Dia) have been implicated in manipulation of the cytoskeleton by HIV. Silencing of Dia1 or its main upstream regulator RhoA impaired infectious synapse progression by reducing actin-dependent HIV polarization and transfer of viral particles from DCs [50], but did not reduce free-virus production from Jurkat cells [91]. On the other hand, Dia2 has been identified as the factor responsible for elongation of HIV-filopodia. HIV filopodia are defined as long (≈10 μm), thin (100 nm), curved membrane projections that are enriched with linear actin filaments and capped with budding HIV virions at their tips. Their occurrence on the surface of infected DCs dramatically enhances the number of intercellular contacts with target T-cells and was observed to promote cell-cell viral transfer in cis [60]. Finally, an immediately recent study revealed critical roles for Dia formins in mediating inbound HIV infection [159]. Interfering with Dia1 or Dia2 dramatically reduced establishment of infection in various cell lines. This was associated with a decrease in HIV-induced microtubule stabilization by Dia, which was shown to assist specific intracellular transport of viral cores towards the nucleus. Furthermore, both formins were found to directly associate with capsid on viral cores, and to promote core-uncoating independently of their cytoskeletal remodeling abilities [159]. Overall, formins seem to play unexpected and diverse but critical roles in HIV infection.

**APC** (adenomatous polyposis coli) is a tumor suppressor gene best known for its roles as a negative regulator in the Wnt signaling pathway and in colorectal cancer [160]. However, APC also belongs to the group of tandem-monomer-binding actin nucleators [161,162,163], and participates in multiple cell functions including cytoskeletal regulation [164,165]. A recent study revealed striking manipulation of APC by HIV. Miyakawa et al. showed that APC interacts with HIV-Gag via its C-terminal domain, which also mediates actin binding [13]. APC expression in 293T cells increased viral production in an actin-dependent manner, whereas APC silencing in HeLa cells decreased targeting of both Gag and viral RNA to the PM and reduced virion infectivity due to impaired incorporation of viral RNA. Furthermore, APC-depletion decreased cell-cell HIV transfer from primary T-cells by reducing localization of viral components to the VS [13]. Thus, APC plays previously unrecognized yet critical roles for outbound HIV spread, and all three classes of actin nucleators are subject to HIV manipulation.

#### 5.1.4. Actin Depolymerizing Factors

Filament depolymerization ensures fluidity of actin pools required for the maintenance of dynamic F-Actin structures [166]. In immune cells, this is particularly important for the formation of membrane protrusions, which mediate cell motility and direct cell-cell interactions [167]. Since impromptu depolymerization is too slow to ensure efficient actin turnover [168], cells have regulators that can bind to actin filaments and mediate their disassembly.

**Cofilin** is a highly homologous protein in eukaryotes and participates in numerous cellular processes [169], thus representing a functional node in cell biology [170]. Many of these functions involve cofilin’s ability to accelerate actin disassembly by two mechanisms; (i) severing of microfilaments; and (ii) enhancing depolymerization rates from both ends [166,171]. Cofilin is a key regulator of the actin cortex, which represents both a barrier and an ally for HIV spread [86]. Thus, manipulation of cofilin by HIV is temporally regulated and involves both exploitation and neutralization at different stages of the viral life cycle [86]. For the sake of clarity, here, we review these events together in the context of inbound HIV. Early binding of Env to CD4 and CXCR4/CCR5 coreceptors triggers rapid Gα signaling and initiates an early wave of actin polymerization that kinetically correlates with a phase of cofilin inactivation [172]. Several findings indicate that this involves multiple Rho-GTPase signaling pathways (Figure 2a). The Rho-GTPase RhoA is activated in a filamin-A-dependent manner, and leads to activation of its downstream effector ROCK1, which in turn phosphorylates and activates LIMK1 [98]. LIMK1 is a serine/threonine kinase that mediates C-terminal phosphorylation of cofilin, resulting in its potent and immediate inactivation [168,173]. On the other hand, Rac1 activation, possibly via Src, leads to cofilin inactivation via the Rac-PAK1-LIMK1 pathway [119]. The resulting drop in cofilin activity allows for a local increase in cortical actin polymerization and the associated capping of CD4/CXCR4 receptors, which promotes fusion at the PM and, thus, facilitates viral entry (Figure 2a).

After virus-cell fusion, intracellular transport of viral components is limited by the dense cortical actin network. Delayed Env-CXCR4 signaling via the receptor-coupled Gαi leads to cofilin activating dephosphorylation, by a mechanism that is not completely understood (Figure 2b) [172]. This results in cortical actin depolymerization that allows passage of the viral core and pre-integration complex to the subcortical cytoplasm on their way to the nucleus (Figure 2c). Indeed, cofilin silencing in primary T-cells leads to aberrant accumulation of cortical actin and reduces HIV integration without affecting viral entry or reverse transcription [172]. Conversely, cofilin activation by different stimuli increased nuclear translocation and HIV infection levels [12,174]. Note that cofilin activation is particularly important for infection of resting CD4+ T-cells, which have predominantly inactive cofilin and low levels of endocytosis [172]. Wu et al. confirmed that resting T-cells from HIV+ patients display higher levels of active cofilin in vivo, with most cells being uninfected [175]. Since these cells can become fundamental components of the HIV latent reservoir, elucidation of the exact mechanism by which inbound HIV leads to cofilin activation remains a critical goal in the field, with potentially major clinical implications. The manipulation of cofilin by outbound HIV is discussed in Section 5.2.2.

**Gelsolin** is one of the most potent actin-severing proteins in the cell [176], but also serves as a versatile modulator of other aspects of actin dynamics [168]. Several findings suggest that HIV exploits gelsolin by strongly inducing its upregulation. Gelsolin protein levels were increased six-fold in PBMCs from HIV-infected patients [177]. A recent clinical study also showed that plasma gelsolin levels were significantly increased in HIV+ individuals [178]. Importantly, this inversely correlated with CD4+ cell counts in subjects before and during treatment. These studies, however, did not reveal whether gelsolin upregulation occurs in infected or uninfected blood cells. Based on previous findings, we hypothesize that gelsolin upregulation occurs in HIV-infected cells by a presently undefined mechanism. Firstly, while modulation of host gene expression in uninfected cells is mainly mediated by Tat [179], expression of Tat alone led to gelsolin downregulation in Jurkat cells [180]. Secondly, overexpression or silencing of gelsolin in T-cell lines impaired inbound HIV infection by promoting excessive loss or gain of cortical actin, respectively, as well as impairment of Env-induced actin remodeling events [181]. Thus, uncontrolled gelsolin upregulation in target cells is unlikely to benefit HIV spread. In summary, gelsolin is strongly induced upon HIV infection and gelsolin plasma levels show clinical predictive value for progression of AIDS. Still, the mechanism(s) by which HIV manipulates this host factor and the underlying reason remain obscure and warrant further study.

### 5.2. Neutralization of Actin Regulators by HIV

Carefully orchestrated manipulation of the actin cytoskeleton by HIV requires not only activation but also neutralization of cellular proteins involved in actin regulation. Neutralization results in a decrease of target protein activity and can be either generalized or spatiotemporally coordinated. Neutralization mechanisms include protein inactivation (direct or indirect), downmodulation, and repression of gene expression. Here, we describe select prominent examples of host cytoskeletal factors that are subject to such manipulation and emphasize the role of these events in facilitating inbound HIV infection and impairment of T-cell immunological functions. It is worth noting that many of the regulators discussed below can also be activated by the virus at other stages of HIV infection.

#### 5.2.1. RhoA

RhoA is another thoroughly studied Rho-GTPase family member and is mainly known for regulating actin stress fiber formation. Unlike Rac1 and Cdc42, RhoA seems to be negatively regulated in HIV-infected cells. In podocytes, HIV infection led to RhoA inhibition and loss of stress fibers [125]. This was shown to be dependent on Nef, via formation of a large multiprotein complex that includes Src and various Rho-GTPase regulators that specifically inhibit RhoA, while promoting Rac1 activation [125,182]. We hypothesize that inhibition of RhoA in HIV-infected cells serves to subdue RhoA-mediated actin remodeling that may antagonize outbound virus spread. This is supported by the findings of several studies. Firstly, Graziano et al. showed that urokinase plasminogen activator inhibits HIV release from macrophages by activation of RhoA [88]. Secondly, overexpression of the Rho-A specific activator p115-RhoGEF (guanine-nucleotide exchange factor) was found to limit HIV-1 replication in 293T and Jurkat cells at the gene expression level and in a RhoA-dependent manner [183]. Furthermore, the cytoplasmic tail of Env was found to interact with a regulatory domain of p115-RhoGEF, thus sequestering it at the PM and preventing RhoA-mediated actin changes. Disrupting this inhibitory interaction by even a single amino acid mutation completely abolished HIV virus production in various T-cell lines [184]. In strong contrast, HIV can induce RhoA activation in cells that are not yet productively infected. Env-CD4 binding was shown to trigger RhoA activation in T-cells [98], whereas interfering with RhoA activity resulted in reduced infection of PBMCs [143]. This is in agreement with the model proposed in Figure 2a. Prominent RhoA activation has also been reported upon Tat treatment in various cell types [124,185,186,187]. In conclusion, Nef and Env likely inhibit RhoA in productively infected cells to neutralize its negative effects on outbound virus release, whereas soluble Tat and inbound Env might activate RhoA in uninfected cells to increase their permissibility to infection.

#### 5.2.2. Cofilin

**Figure 4:**
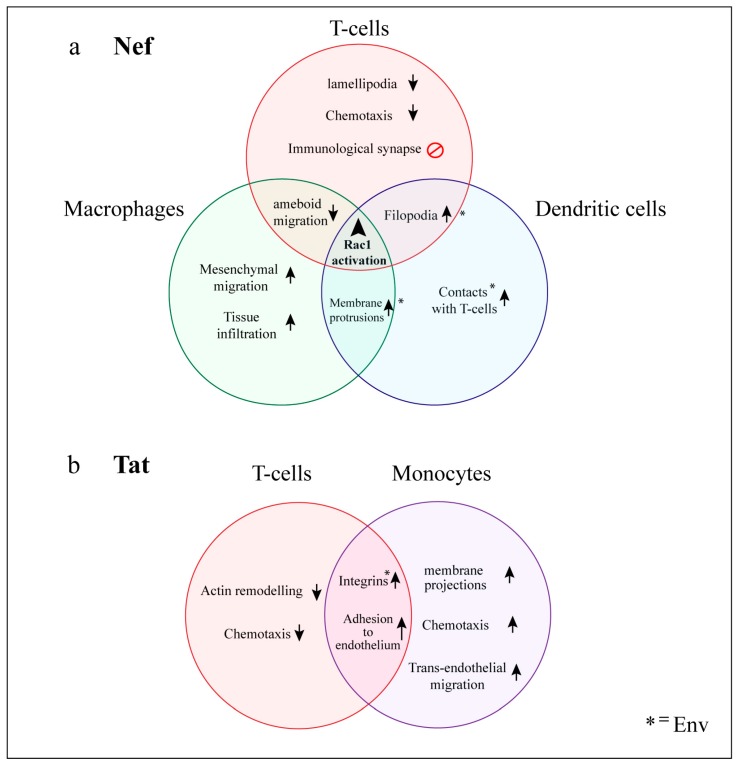
Actin-related changes in cellular behavior induced by HIV in relevant cell types. Coordinated manipulation of actin regulators results in global and cell-type specific changes in cellular morphology and motility that contribute to viral spread, impairment of immune function and HIV comorbidities. Many of these changes can be mapped to specific HIV accessory proteins, which act as master regulators of the cytoskeleton. Nef and Tat are expressed in HIV-infected cells but are also present extracellularly in serum and, thus, can affect both infected and uninfected cells. (**a**) Nef; leads to Rac1 activation in a wide range of cell types. In T-cells, this is associated with inactivation of Cofilin and severe cytoskeletal disorganization, which impairs cell migration and immunological synapse formation. In myeloid cells, Nef enhances formation of several membrane protrusions which promote cell motility and contacts with uninfected cells; (**b**) Tat modulates the expression of numerous genes involved in actin regulation. In T-cells, Tat interferes with chemotaxis and F-actin remodeling, whereas in monocytes it increases cell motility, chemotaxis and phagocytosis. Tat also induces expression of adhesion molecules and promotes leukocyte binding to the endothelium. Upward arrows represent enhancement of biological processes or increases in number of structures, whereas downward arrows represent impairment of processes. * = Effects also induced by HIV envelope glycoprotein (Env).

In contrast to the fine-tuned manipulation of cofilin by inbound HIV via Env (Figure 2), productively infected cells seem to display a broad neutralization of cofilin activity, which is mainly mediated by Nef. Expression of Nef led to potent cofilin inactivation in both 293T and primary T-cells [188,189]. Importantly, this feature of Nef is strongly conserved across multiple strains of HIV-1, HIV-2 and simian immunodeficiency virus (SIV) [190], which suggests a critical advantage of cofilin neutralization upon productive infection. Indeed, Nef-mediated cofilin inactivation is associated with dramatic impairment of actin remodeling, cell migration and immunological functions in infected T-cells (Figure 4a). The mechanism of inactivation involves phosphorylation by cellular kinases hijacked by Nef (see Section 5.3.2). Furthermore, Tat expression was shown to decrease total cofilin protein levels in Jurkat cells [180]. While multiple neutralization strategies suggest a negative role of cofilin for outbound virus, several findings argue against this view. Firstly, cofilin was consistently incorporated in virions produced by myeloid and lymphoid cells [73,102], reaching up to 10% of the molar abundance of Gag [69]. Secondly, silencing of cofilin or its upstream regulator LIMK1 led to a ~5-fold reduction in free-virus production by HeLa cells, which was associated with significant accumulation of nearly mature HIV particles on the PM, suggesting impairment of the final release step [19]. How these observations fit with broad cofilin inactivation remains to be resolved. One possibility is that cofilin contributes to outbound virus independently of its ability to reorganize actin. Alternatively, cofilin may be differently manipulated in diverse cell types. Cofilin inactivation was not detected in infected macrophages, despite clear occurrence of other Nef-mediated actin effects [191]. Overall, the abilities of multiple HIV proteins to neutralize the depolymerizing factor cofilin emphasize the key role that microfilaments play at various stages of the viral life cycle.

#### 5.2.3. N-WASP

N-WASP is a ubiquitously expressed NPF that stimulates Arp2/3 activity downstream of Rac1, and participates in regulation of actin dynamics at the PM [158]. In T-cells, Nef potently interferes with actin polymerization changes downstream of engaged T-cell-receptors (TCR), and these effects can be mimicked by pharmacological inhibition of N-WASP [192]. Indeed, Nef was found to impair activating phosphorylation of N-WASP early upon TCR-signaling, and its later recruitment to the immunological synapse (see Section 6.1.1). The mechanisms by which Nef interferes with N-WASP activity are poorly understood, but are known to be dependent on Nef’s SH3-binding motif, thus suggesting involvement of a yet unidentified cellular kinase [192].

### 5.3. Actin Regulators Hijacked by HIV

Some of the best understood examples of cytoskeletal manipulation are given by cases where regulators of actin dynamics are directly engaged by viral factors via protein–protein interactions. We consider such host factors to be hijacked by HIV whenever their activation results as a direct consequence of physical interaction with a viral protein. Importantly, this allows the virus to override cellular regulatory pathways, by promoting downstream events even in the absence of upstream activating signals, or despite the presence of inhibitory cues. In addition, our definition of hijacking includes manipulation events where the functional outcome of host protein activity is altered, for example, by modification of host protein interaction partners, and/or deliberate alteration of protein subcellular localization. Since most (if not all) of the cytoskeletal exploitation and neutralization events likely occur downstream of HIV-hijacked host factors, resolving the molecular mechanisms by which the virus engages these proteins is of essential importance to the field.

#### 5.3.1. Vav

Vav is a proto-oncogene predominantly expressed in hematopoietic cells and serves as a guanine nucleotide exchange factor (GEF) and potent activator for both Rac1 and Cdc42. Nef engages Vav directly, and this results in strong Vav activation [141,193]. While the activation mechanism is incompletely understood, it is known to require Nef’s proline-rich motif (PxxP) binding to the SH3 domain of Vav, and likely involves (i) conformational changes; (ii) phosphorylation of Vav via recruitment to multiprotein complexes with cellular kinases such as Src [125,129]; and (iii) direct targeting of Vav to specialized PM microdomains that contain its downstream effectors Rac1, Cdc42 and PAK2 [194]. Both Vav activation and its GEF-ability are required for Nef-induced activation of Rac1/Cdc42 and the associated actin remodeling changes [129,141]. In agreement, silencing of Vav reduced the positive effect of the Rac-Wave2-Arp2/3 pathway on outbound viral release, but only for Nef+ virus [91]. Furthermore, various lines of evidence suggest that Vav is required for Nef-mediated PAK2 activation [141,194,195], despite the fact that Nef binds PAK2 directly and activates Rac1 and Cdc42, both of which act upstream of PAK2. Therefore, since a large proportion of cytoskeletal manipulation events by outbound HIV are dependent on Vav, strategies that prevent Nef-mediated Vav activation could have promising therapeutic potential.

#### 5.3.2. PAK

The existence of a “Nef-associated kinase” was recognized as early as 1994 [196]. Subsequent studies showed that this serine/threonine kinase activity corresponds to the “p21-activated kinases” PAK1 and PAK2. PAKs are important Rho-GTPase downstream effectors that regulate numerous cytoskeletal functions [197]. Nef binds directly to PAK1 and PAK2 [198,199], and thereby promotes important cytoskeletal changes downstream of Rac1 and Cdc42 [198,199,200,201,202]. Despite intensive study and identification of the Nef domains required for this interaction [202], the mechanism for Nef-mediated PAK activation is still incompletely understood. It is thought to involve autophosphorylation [203], and targeting to membrane lipid-rafts, where it meets Vav, Rac1 and Cdc42 [131,194,195,204,205]. The in vivo relevance of this activation was highlighted by a study looking at the infection of macaques with SIV. Shortly after inoculation, Nef point-mutants incapable of PAK activation revert to original Nef function and sequence, preceding increases in viral load and disease progression [206]. This indicates a strong selective pressure for functional Nef:PAK interaction in the infected host. A similar study confirmed these in vivo results and further supported a role for Nef-PAK2 during stages of chronic infection [207]. Functionally, Nef-mediated PAK2 activation increases outbound virus production [131], and results in deregulation of the actin-depolymerizing factor cofilin. Stolp et al. showed that Nef not only activates PAK2 but also modifies its specificity for cofilin, which is not normally a PAK2 substrate [25]. Thus, Nef hijacks PAK2 to induce hyperphosphorylation of cofilin and interferes with actin remodeling in infected T-cells [190] (Figure 4a). A recent study also revealed that Nef exploits PAK2, independent of its kinase activity, to interfere with actin remodeling downstream of TCR-signaling [208]. Overall, the ability to hijack PAKs is a highly common feature of Nef [190,209], and plays a critical role for outbound virus spread and disease progression.

#### 5.3.3. Hck

The hematopoietic cell kinase (Hck) is a member of the Src family and is expressed selectively and at high levels in phagocytic cells, including macrophages, monocytes and DCs [191,210], where it regulates various inherent functions of these cells that involve actin polymerization [211]. Manipulation of Hck by HIV is dependent on Nef, and thus mainly affects infected cells. Nef binds Hck directly and with much higher affinity than other SH3-bearing kinases [212,213]. This dramatically increases Hck activity by inducing its autophosphorylation and by releasing the catalytic region via displacement of the SH3 domain [210,214]. The crystal structure of the complex has been resolved up to 1.8 angstroms, confirming engagement of the Hck-SH3 domain by Nef’s PxxP motif, and suggesting that Hck conformational changes might reciprocally reorganize Nef to promote interaction with other signaling partners [215].

In macrophages, HIV infection was shown to increase already high levels of Hck expression and activity. This correlated with increased virus production, whereas Hck silencing strongly impaired viral release [216]. In agreement, disruption of the Nef:Hck interaction was shown to impair viral replication [212,217]. Furthermore, Hck was found within virions produced by macrophages and Hck-expressing 293T cells. Incorporation was dependent on Nef’s PxxP domain, suggesting that loading into the virus occurs by direct binding to Nef [218]. Expression of Hck in producer cells also increased particle infectivity [218], whereas Hck silencing decreased it at the entry level [219]. Importantly, Nef reprograms macrophage migration via Hck by inducing dramatic cytoskeletal changes [191]. Nef increased the number, size, F-actin content and life-span of podosome structures on the surface of infected macrophages in a Hck-dependent manner. This is likely a consequence of strong WASP activation by Hck, given the massive accumulation of phosphorylated WASP on these structures [191]. Nef also increased adhesion, matrix degradation and mesenchymal migration, while inhibiting ameboid migration of infected macrophages. In *nef*-transgenic mice, this results in macrophage tissue accumulation that mimics that of HIV+ patients [191]. A similar in vivo study showed that Hck is required for rapid onset of Nef-induced AIDS-like disease. Both Hck-knockout mice and those with mutations in the Hck-SH3 domain displayed prolonged latency periods and delayed appearance of symptoms [220]. Altogether, Hck is subject to profound and multifaceted manipulation by HIV that includes upregulation, strong direct activation and virion incorporation. This enhances Hck actin remodeling and promotes outbound virus spread by increasing both virion release and infectivity. Furthermore, current evidence indicates a strong role for Nef:Hck in HIV pathogenesis in vivo, and highlights Hck as an optimal target for pharmacological intervention in the clinic. The nature of different Hck inhibitors and their potential as therapeutic agents for HIV infection is of great current interest in the field and has been recently reviewed in [221].

## 6. Functional Consequences of HIV Manipulation of Actin Networks

Like most retroviruses, HIV is constrained by its genome size. A common theme of HIV infection, like many other viral infections, is to utilize and manipulate as many cellular pathways to its benefit as possible, through expression of a limited number of viral proteins. For HIV, this must take place in the context of the landscape of our immune system, especially in CD4+ T-cells and CD4+ antigen presenting cells, including macrophages and DCs. Immunological tropism and systematic manipulation by HIV inevitably affect the host’s immune system at many levels. Viral manipulation in the form of actin remodeling is not only key to promoting spread of viral infection, but also compromises normal immunological functions. Three decades of research have increased our understanding of how HIV collectively reprograms actin network dynamics in these cells to promote specific changes in cellular behavior. In this section, we discuss the functional consequences of global actin cellular changes induced by HIV and highlight the striking differences in morphology and motility observed between infected lymphoid and myeloid cells (Figure 4).

### 6.1. T-cells

In CD4 T-cells, HIV infection and expression of viral proteins are associated with compromised immune functions [222]. This is, in part, due to dramatic deregulation of actin remodeling, which severely impairs cell motility. Nef plays a major role in this process, and expression of this viral protein alone is sufficient to impair chemotaxis, cell movement and transendothelial migration both in vitro and in vivo [223,224,225,226]. This is associated with the observed impairment of membrane protrusions [225], which is likely explained by strong aberrant activation of Rac1/Cd42 [227], and simultaneous neutralization of cofilin via PAKs [189]. Nef also interferes with chemokine receptor signaling involved in cell migration [192,223]. In addition, Tat mediates important cytoskeletal changes that affect T-cell immune function, mainly by modulation of host gene expression. Tat consistently downregulates multiple actin regulatory proteins including cofilin and gelsolin [180], whereas it increases RhoA activity by upregulating several of its upstream activators [187]. Together, these changes lead to an aberrant increase in F-actin content that in T-cells translates into impaired chemotaxis and cell migration [187,228]. Interruption of chemotaxis likely increases local viral spread within lymphoid tissue, as commonly observed in AIDS patients.

#### 6.1.1. Impairment of the Immunological Synapse

HIV-1 also disrupts effective communication of the immune response from infected T-cells by blocking formation of the immunological synapse (IS) [229,230], which is a highly organized multimolecular structure that forms at the junction between T-cells and antigen presenting cells during antigen presentation [231]. Most detrimental effects to the IS are mediated by Nef [232]. Nef-mediated inhibition of N-WASP prevents actin remodeling changes required for cell-spreading, cell-cell contacts and actin-ring formation, but also interferes with recruitment/activation of the TCR signaling machinery [192]. In addition, Nef can bind to Lck directly [233], and induces intracellular accumulation of TCR and Lck in endocytic compartments [234]. This not only explains their decreased accumulation at the IS [234,235], but also the reduced phosphorylation of their associated downstream signaling proteins Zap70 and Lat [192]. While TCR-signaling components are not cytoskeletal regulators per se, they are known to trigger actin remodeling upon TCR activation [236]. Overall, Nef employs diverse mechanisms to impair actin remodeling changes required for IS formation [237].

#### 6.1.2. Virological Synapse Formation

While HIV seems to selectively interfere with actin-dependent structures that are required for normal T-cell function, it readily allows formation of those that promote cell-cell transfer of HIV. Virological synapse formation involves complex rearrangement of host and viral molecules, and while this occurs in a strongly actin-dependent manner [17], it is not negatively affected by Nef [238]. Of note, the coordinated deregulation and relocation of many actin-regulatory proteins on both the donor- and target-cell sides, culminating in the formation of a specialized viral transfer structure, represents one of the most complex forms of cytoskeletal manipulation known to date. While the exact mechanisms and hierarchy of molecular events has not yet been fully resolved, here, we summarize some of these events in the context of the T-cell VS. Jolly et al. found that VS formation is strongly dependent on initial binding of Env and CD4 [48]. Env likely induces the activation of the integrin LFA-1 on the target-cell side [239,240], increasing its ligand affinity and leading to binding of ICAM-1 on the donor-cell side, which is important for intercellular conjugate formation [17,241]. Env-CD4/CXCR4 and LFA-1 signaling also lead to actin remodeling and actin-dependent polarization of LFA-1, CD4 and CXCR4 on the target-cell side. This likely requires anchoring of these molecules to actin filaments by membrane-cytoskeletal linkers such as filamin-A, ERMs and focal adhesion proteins. Indeed, Talin was observed to colocalize with LFA-1, often forming a “ring-like structure” [48]. Polarization of Env on the donor-cell side is dependent on CD4 clustering on the target cell, and/or actin-remodeling on the donor cell, and is accompanied by polarization of Gag [48]. Other cellular components are also polarized to the donor-cell side in an actin- or tubulin-dependent manner, including phosphorylated ERMs [110], tetraspanins [242], the microtubule organization center [243], and mitochondria [244]. Recent studies have revealed that LFA-1 [245], as well as the signaling molecules ZAP70, Lck and the TCR are involved in some of these processes [139,245,246]. Donor cell polarization ultimately results in polarized viral assembly, budding and highly efficient transfer of virus to the target cell across the synaptic space [31,48,54,247,248,249]. Overall, by blocking immunological synapse formation and promoting virological synapses, the virus reprograms the T-cell cytoskeleton to communicate HIV instead of communicating the immune response.

### 6.2. Myeloid Cells

In contrast to T-cells, infected myeloid cells tend to display aberrant enhancement of actin-rich membrane protrusions. This can result in increased cell motility, altered cell-matrix adhesion or intercellular contacts, shifts in tissue distribution, and other changes that compromise normal immune function while promoting viral spread (Figure 4). In macrophages, HIV increases the number and size of actin-rich podosomes, which correlates with strongly enhanced mesenchymal migration and tissue infiltration. This requires Hck manipulation by Nef, whereas impairment of cofilin regulation was not observed [191]. In addition, Tat impairs macrophage phagocytosis by preventing recruitment of Cdc42 to the phagocytic cup [250]. In monocytes, Tat induced integrin expression [251,252], and increased adhesion to the extracellular matrix and endothelium [251,253,254], as well as transendothelial migration [254,255]. Soluble Tat also induced thin actin-rich membrane protrusions [251], and promoted chemotaxis of monocytes, neutrophils and macrophages [228,254,256]. Note that increased tissue infiltration and impaired phagocytosis in both macrophages and monocytes have been observed in HIV+ patients in vivo, and this is associated with various aspects of disease [257]. In dendritic cells, both Nef and Env induce actin remodeling changes that promote formation of HIV-filopodia and thicker membrane extensions, respectively. While structurally different, both types of actin-rich membrane protrusions increase intercellular contacts with T-cells, and facilitate cell-cell viral transfer either in cis or in trans, respectively [50,60]. Exogenous Nef also triggers DC ruffle and uropod formation [129], whereas soluble Env induces DC chemotaxis and migration by a specific actin polymerization pathway [258,259].

Altogether, HIV can manipulate actin dynamics to promote tissue infiltration of infected monocytes/macrophages and enhance migration of both infected and uninfected dendritic cells. These actin-dependent changes likely reinforce the role of myeloid cells as potentially essential components of the viral reservoir in different organs, and in viral dissemination via direct cell-cell contacts. Often overlooked in terms of HIV infection, antigen presenting cells work in small numbers to drive the immune response, and thus small numbers of these cells are equally capable of spreading HIV infection. Since communication of the immune response requires highly coordinated and dynamic changes in their cytoskeleton, it is unsurprising that coordinated manipulation of the actin cytoskeleton is also required in these cells to mediate viral spread.

## 7. Conclusions and Perspective

Several decades of research have illuminated numerous links between HIV and components of the actin cytoskeleton. On one hand, this has revealed a wide range of strategies and targets that the virus is capable of using to manipulate cellular actin networks. On the other hand, it has increased our understanding of how these changes collectively modify cellular processes that promote positive infection outcomes. However, it is important to keep in mind that the findings reviewed here derive from a multitude of studies employing different experimental approaches, model cell systems and research questions. This heterogeneity and the occasional lack of corroboration in the documented observations therefore make it difficult to fit all available data into comprehensive hypothetical models. In addition, it should not be automatically assumed that all reported manipulation events take place in vivo, or that they are of importance to the virus.

It is our suggestion that future efforts should aim to expand our understanding of those HIV-modulated pathways that resonate across multiple cell types, as well as those which can be linked to cellular changes that increase viral infection, persistence or transmission. Despite recent outstanding efforts and creditable advances in this context, several critical issues remain unresolved. These include, but are not limited to: (i) the exact mechanisms by which Rho-GTPases are activated downstream of chemokine coreceptors to reorganize cortical actin and promote inbound HIV infection; (ii) the mechanisms by which proteins that directly mediate actin remodeling (such as Arp2/3, APC and cofilin) enhance outbound virus release and/or cell-cell transfer; (iii) the role of actin regulators that are specifically incorporated into HIV particles; (iv) the spatiotemporal events that allow recruitment of cytoskeletal proteins to virological synapses, as well as the role that they play in these structures; and (v) the key manipulation events that allow differential modulation of leukocyte migration patterns in lymphoid and myeloid cells. In addressing these questions, the field would greatly benefit from more consistent use of defined and well-controlled methodologies, specifically designed to evaluate the role of actin regulators at different stages of the viral life cycle. Systematic studies that allow comparison of the effects of multiple regulators and their mapping to HIV-manipulated pathways would represent the gold standard in this setting. Furthermore, these efforts should be focused on cell types that are physiologically relevant for HIV infection, and where possible, validate significant findings in primary cells and/or animal models (where it is logistically and ethically possible). The power of new high-resolution imaging techniques, genetic manipulation approaches and library screening capacities will undoubtedly catalyze advances in our quest of resolving the intersection between HIV and the actin cytoskeleton. Overall, the evidence obtained so far suggests that interplay with the actin cytoskeleton occurs at nearly all stages of the viral life cycle. However, targeted manipulation of actin regulators and pathways is particularly important for promoting viral entry, inbound intracellular transport, and highly efficient viral spread via direct cell-cell contacts.

Considering that the study of pathogens and actin has previously contributed greatly to our understanding of the cytoskeleton, HIV represents a unique opportunity to dissect specialized cytoskeletal pathways that are specific to cells from our immune system. Furthermore, the development of therapeutic strategies that target host factors which are exploited by pathogens is a highly attractive goal in modern medicine, since it dramatically reduces the chance of drug resistance. Ultimately, the development of cytoskeletal-modulating drugs also has overreaching clinical potential in the treatment of other diseases that involve deregulation of cytoskeletal proteins, including various neurological disorders, cardiovascular disease and cancer.
